# The Role of Oxidative Stress in Trisomy 21 Phenotype

**DOI:** 10.1007/s10571-023-01417-6

**Published:** 2023-10-11

**Authors:** Angelika Buczyńska, Iwona Sidorkiewicz, Adam Jacek Krętowski, Monika Zbucka-Krętowska

**Affiliations:** 1 Clinical Research Centre, Medical University of Białystok, ul. M. Skłodowskiej-Curie 24a, 15-276 Białystok, Poland; 2 Department of Endocrinology, Diabetology and Internal Medicine, Medical University of Białystok, ul. Sklodowskiej-Curie 24a, 15-276 Białystok, Poland; 3 Department of Gynecological Endocrinology and Adolescent Gynecology, Medical University of Białystok, ul. M. Skłodowskiej-Curie 24a, 15-276 Białystok, Poland

**Keywords:** Trisomy 21, Oxidative stress, Antioxidant capacity, Cognitive impairment, Lipid peroxidation, Mitochondrial dysfunction

## Abstract

Extensive research has been conducted to gain a deeper understanding of the deregulated metabolic pathways in the development of trisomy 21 (T21) or Down syndrome. This research has shed light on the hypothesis that oxidative stress plays a significant role in the manifestation of the T21 phenotype. Although in vivo studies have shown promising results in mitigating the detrimental effects of oxidative stress, there is currently a lack of introduced antioxidant treatment options targeting cognitive impairments associated with T21. To address this gap, a comprehensive literature review was conducted to provide an updated overview of the involvement of oxidative stress in T21. The review aimed to summarize the insights into the pathogenesis of the Down syndrome phenotype and present the findings of recent innovative research that focuses on improving cognitive function in T21 through various antioxidant interventions. By examining the existing literature, this research seeks to provide a holistic understanding of the role oxidative stress plays in the development of T21 and to explore novel approaches that target multiple aspects of antioxidant intervention to improve cognitive function in individuals with Down syndrome.

**Graphical Abstract:**

The guides -base systematic review process (Hutton et al. 2015).
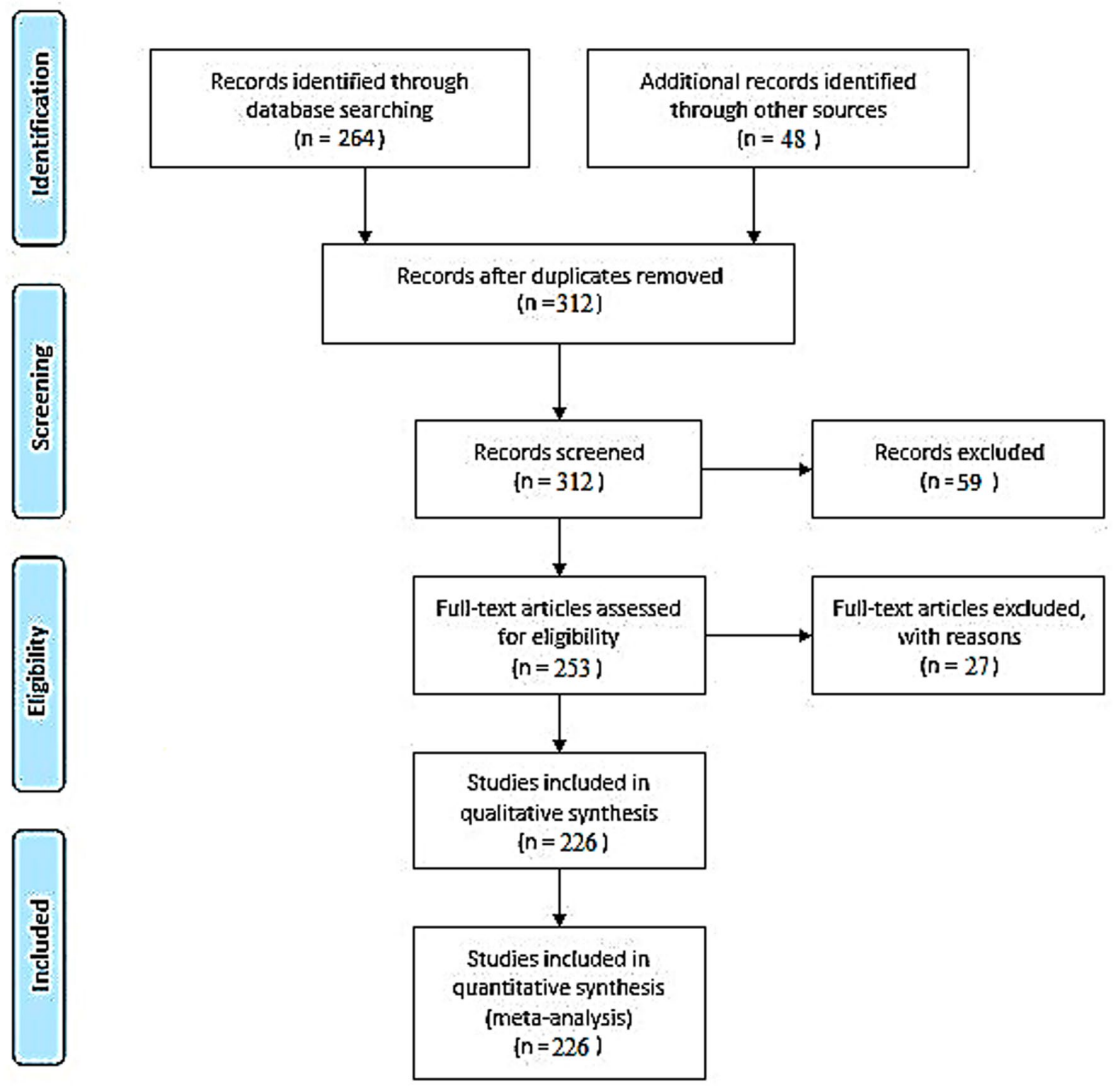

## Introduction

Trisomy 21 (T21), also known as Down syndrome (DS), is the most prevalent genetic disorder affecting fetuses (Sherman et al. 2007; de Graaf et al. 2021; de Graaf et al. 2017; Murthy et al. 2007). T21 occurs at an incidence of approximately 1 in 700 to 1 in 1000 births worldwide (Irving et al. 2008; Mai et al. 2019). It is characterized by the presence of an extra copy of chromosome 21 and can result from various genetic abnormalities such as maternal non-disjunction, mosaic karyotype, Robertsonian translocation, or partial duplication of the critical region of chromosome 21(Hoeffer et al. 2007; Antonarakis et al. 2020; Akhtar and Bokhari 2021). Individuals with T21 exhibit distinctive features including cognitive impairment, muscle hypotonia, and dysmorphic characteristics (Lana-Elola et al. 2011; Korenberg et al. 1994; Korbel et al. 2009). Proper diagnosis and treatment of neurological complications are essential for optimizing the life expectancy and well-being of individuals with DS (Lott and Dierssen 2010). Given the multiple impairments associated with the presence of an additional chromosome 21, it is crucial to gain novel insights into the disrupted metabolic pathways, particularly those related to cognitive impairment improvement (Narasimhan et al. 2013; Jackson et al. 2019; Adelekan et al. 2012; Whooten et al. 2018; Hartley et al. 2015; Gardiner 2014). Understanding the functions of genes on chromosome 21 and their impact on cognitive impairment is of paramount importance (Ishihara and Akiba 2017). Gene mapping studies have identified lipid peroxidation, mitochondrial dysfunction, and impaired neurogenesis as potential factors contributing to the neuronal phenotype and cognitive impairment in DS. Recent clinical investigations have highlighted a connection between oxidative stress and cognitive deficits in individuals with T21 (Zis et al., n.d.; Lott 2012; Ishihara and Akiba 2017; Zafrilla et al. 2014). Consequently, there has been discussion regarding antioxidant interventions aimed at reducing the negative consequences of increased oxidative stress, such as lipid peroxidation, mitochondrial dysfunction, and disturbances in neurogenesis factor synthesis (Picca et al. 2020). Despite these findings, there is currently a lack of clinical management strategies or the introduction of novel treatment modalities specifically for T21 (Muchová et al. 2014). Consequently, multidirectional target analyses are still being explored, along with subsequent evaluations of their potential as DS biomarkers and therapeutic targets. Further research is needed to better understand the underlying mechanisms and to develop effective interventions to improve cognitive function and overall outcomes in individuals with T21.In this review, we examine the available data on the DS phenotype and highlight the emerging role of oxidative stress in its pathogenesis. Furthermore, we compare various antioxidant interventions that have been employed to address DS-related cognitive impairment, along with subsequent analysis of clinicopathological features. The review aims to shed light on the relationship between oxidative stress and the DS phenotype by examining relevant studies. It explores how oxidative stress may contribute to the cognitive impairments observed in DS and investigates different antioxidant strategies that have been utilized to mitigate these impairments. Through a comprehensive analysis of the literature, we assess the effectiveness of various antioxidant implementations in improving cognitive function in individuals with DS. This includes a consideration of the clinical and pathological features associated with these interventions, providing insights into their potential benefits and limitations. By summarizing and comparing the existing research, this review seeks to enhance our understanding of the interplay between oxidative stress and the DS phenotype, as well as provide valuable information on antioxidant approaches for addressing cognitive impairment in DS.

## Materials and Methods

A systematic literature review was performed utilizing the PubMed database, adhering to the guidelines set forth by the Preferred Reporting Items for Systematic Reviews and Meta-Analyses (PRISMA) and the Enhancing the QUAlity and Transparency Of health Research (EQUATOR) network (Hutton et al. 2015; Schiavo 2019). The review was conducted in accordance with a registered protocol in the PROSPERO database, with the registration number CRD42022302440. By following the PRISMA and EQUATOR network guidelines, as well as registering the review protocol in PROSPERO, this literature review strives to ensure a rigorous and transparent approach to synthesizing and analyzing the available evidence related to the research question or topic of interest.

The literature search was performed in February 2022, utilizing specific keywords related to the research topic. The keywords included trisomy 21, Down syndrome, oxidative stress, antioxidant capacity, lipid peroxidation, mitochondrial dysfunction, serine/threonine protein kinase, neurogenesis, antioxidant treatment, and clinical trials. During the review process, manuscripts that exhibited poor study design, lack of clearly defined eligibility criteria, irrelevant definition of exposures, contrasts, and outcomes, outdated studies, inconsequential characteristics of study participants, inadequate reporting of results, or a plurality of the study group were excluded. By applying these inclusion and exclusion criteria, the review aimed to ensure the inclusion of relevant and high-quality studies that provide valuable insights into the role of oxidative stress, antioxidant interventions, and associated clinical trials in trisomy 21 or Down syndrome. The rigorous evaluation of the selected manuscripts helps to maintain the integrity and validity of the review findings.

## Mapping Chromosome 21: Is it an Indication for the Oxidative Status Research?

The T21 is considered a comprehensive and reliable model for conducting experimental studies on genotype–phenotype relationships (Salehi et al. 2007). Chromosome 21 harbors multiple genes that have been associated with Down syndrome (DS) phenotypes. These genes include Cu/Zn superoxide dismutase (SOD1), amyloid precursor protein (APP), Ets-2 transcription factors, Down Syndrome Critical Region 1 (DSCR1), stress-inducible factor, beta-secretase 2 (BACE2), and S100 calcium binding protein B (S100B) (Coskun and Busciglio 2012; Zewen Liu et al. 2017a, b, c)In addition, an aberrant gene expression profile is linked to the mitochondrial phenotype observed in DS, characterized by reduced respiratory efficiency, changes in mitochondrial morphology, altered oxidative metabolism, and impaired mitochondrial biogenesis (Izzo et al. 2017; Sobol et al. 2019) (Table 1).

**Table 1:** Genes associated with oxidative stress characteristics in DS

Gene	Name	Protein significance	References
Cause of oxidative status disturbance in DS			
*DYRK1A*	Dual Specificity Tyrosine Phosphorylation Regulated Kinase 1A	A member of the Dual-specificity tyrosine phosphorylation-regulated kinase (DYRK) family involved in promotion of apoptosis in response to oxidative stress	Choi and Chung (2011) and Kimura et al. (2006)
*NRIP1*	Nuclear Receptor Interacting Protein 1	Interaction with the hormone-dependent activation of nuclear receptors (modulation of transcriptional activity of the estrogen receptor)	Izzo et al. (2014)
*SOD1*	Superoxide Dismutase Type 1	Mitochondrial protein responsible for destroying free superoxide radicals	Netto et al. (2004)
*SUMO3*	Small Ubiquitin Like Modifier 3	Involved in nuclear transport, DNA replication and repair, mitosis, transcriptional regulation, and signal transduction	Prandini et al. (2007)
The effect of impaired oxidative status in DS			
*CBS*	Cystathionine Beta-Synthase	Involved in mitochondrial dysfunction based on impaired oxidative stress detoxification by either oxidation catalyzed by aldehyde dehydrogenase (ALDH) or by reduction to their corresponding alcohols by carbonyl reductase (CBR) and/or alcohol dehydrogenase (ADH)	Marechal et al. (2019) and Perluigi and Butterfield (2012)
*S100B*	S100 Calcium Binding Protein B	Cell cycle regulator	Lu et al. (2011), Whitaker-Azmitia et al. (2010)

Emerging evidence indicates that gene therapy holds promise as a potential approach to modulate enzymatic activities and counteract the detrimental effects of oxidative stress. This therapeutic strategy has shown successful outcomes in various neurodegenerative and neurological disorders (X. Zhu et al. 2021; Sun and Roy 2021). Mounting evidence supports the notion that disturbances in neurogenesis and oxidative stress play a significant role in the development of diverse neuronal abnormalities observed in individuals with DS (Walton et al. 2012; Nunomura et al. 2000; Helguera et al. 2013; Pagano and Castello 2012).

## Oxidative Stress

A connection has been established between oxidative stress and neuronal cell apoptosis in the development of numerous neurodegenerative disorders (Fujita et al. 2012). The accumulation of reactive oxygen species (ROS) disrupts cellular signaling pathways and is linked to brain injury and the progression of neurodegenerative conditions (T.-F. Yuan et al., n.d.; Nunomura et al. 2000; Iannello et al. 1999). The presence of oxidative stress causes the impairment of the blood–brain barrier, depolarization of membranes, inhibition of membrane enzyme activity, and inadequate protein transport (Singh et al. 2019). The reduction in antioxidant capacity, along with impaired neurogenesis, has the potential to exacerbate nerve damage (Timme-Laragy et al. 2013).

Consequently, the supplementation of antioxidants in individuals with DS has the potential to offer protection against the progression of neuronal dysfunction. Promising findings have emerged from studies focusing on enhancing psychomotor development through the use of antioxidant interventions, particularly those based on phytochemical compounds derived from plants. These plant-derived alternative antioxidants (AOX) encompass various dietary phytochemicals such as polyphenols, quinones, flavonoids, catechins, coumarins, and terpenoids, which are considered crucial exogenous AOX.

Previous research has already demonstrated the ability of AOX administration to impede the advancement of neurodegenerative diseases. However, the precise mechanism of action of AOX has yet to be fully elucidated. Ongoing investigations are aimed at further understanding the specific ways in which AOX exert their protective effects in DS and other related conditions (Kumar and Khanum 2012). The existence and characterization of receptors or transporters for phytochemicals in brain tissues have yet to be definitively established, and the outcomes of clinical trials investigating the application of AOX may yield promising results for individuals with T21. Consequently, therapeutic options that encompass compounds with multiple targets are deemed viable for addressing diseases with multifactorial causes, such as DS (Baburamani et al. 2019).

In recent years, there has been a substantial body of research highlighting the neuroprotective potential of phytochemicals and antioxidants, as well as their positive impact on the prevention of neurodegenerative disorders (Gandhi and Abramov 2012; Perrone et al. 2007; Barone et al. 2017; Kurabayashi et al. 2019). The assessment of oxidative stress in DS facilitates a better comprehension of the underlying mechanisms contributing to cognitive impairment, thereby driving the exploration of novel and promising molecular targets for elucidating and mitigating neurodegeneration, starting from early human prenatal development (Yadav et al. 2022).

### Human Prenatal Development

Perluigi et al. conducted a study that focused on analyzing triplicated chromosome 21, which contains several genes associated with oxidative stress. The researchers evaluated various oxidative stress markers, including protein carbonylation, protein-bound 4-hydroxy-2-nonenal (HNE), levels of total and oxidized glutathione (GSH), concentrations of heat shock protein (HSP) and thioredoxin (TRX), in amniotic fluid samples. The investigation utilized techniques such as slot-blot analysis, enzymatic assays, and Western blot. The study findings demonstrated that oxidative stress occurs early in pregnancies affected by trisomy 21 (T21), as evidenced by increased protein oxidation, lipid peroxidation, reduced levels of GSH and TRX, and the induction of the HSP response. These processes have been implicated in the DS phenotype, providing insights into the role of oxidative stress in T21 pathogenesis (Perluigi et al. 2011; Zis et al., n.d.; Slonim et al. 2009; Marcovecchio et al. 2021; Convertini et al. 2016). The oxidative stress markers were also assessed following the collection of maternal plasma and amniotic fluid samples from confirmed T21 pregnancies (Barone et al. 2018; Buczyńska et al. 2021; Pietryga et al. 2021). The study results revealed a significant increase in DNA/RNA oxidative stress damage products in amniotic fluid samples from pregnancies affected by T21 compared to euploid samples. However, no significant difference was observed in plasma measurements between the two groups (Buczyńska et al. 2021). In pregnancies affected by T21, the concentration of advanced glycation end products (AGE) was found to be reduced in both plasma and amniotic fluid samples compared to euploid pregnancies. Conversely, the antioxidant marker asprosin exhibited significantly higher concentrations in both plasma and amniotic fluid samples within the T21 group when compared to euploid pregnancies. The therapeutic mechanism of asprosin is believed to be associated with its beneficial effects on insulin resistance, oxidative stress, and neuropathy (M. Wang et al. 2019; Ozcan et al. 2022). Furthermore, the findings indicated that the concentrations of alpha-1-antitrypsin (A1AT) and 25-hydroxy vitamin D (25-OH vitamin D) were downregulated in cases of trisomy 21 (T21) aneuploidy. This suggests that the decreased A1AT concentration, coupled with exacerbated inflammatory processes and heightened oxidative stress observed in T21 pregnancies, may have a negative impact on multiple comorbidities, potentially contributing to the development of the DS phenotype (Buczyńska et al. 2021). The findings from this study suggest a hypothesis that oxidative stress primarily occurs within the fetus affected by T21 rather than in the maternal compartment. Additionally, it is proposed that the maternal antioxidant mechanisms may be inadequate to compensate for the antioxidant deficiencies induced by the developing T21 fetus (Buczyńska et al. 2021). It is crucial to emphasize that the impact of oxidative stress has already been observed during prenatal development. Therefore, when considering potential medications aimed at reducing oxidative stress, it is essential to ensure not only their effectiveness but also their safety for the developing fetus (Scott et al. 2020; Buczyńska et al. 2021; 2020).

### T21 Childhood

In a following study conducted by Zitnanova et al., the concentration of oxidative stress markers was assessed using high-performance liquid chromatography (HPLC). The study revealed that the group of children with T21, consisting of 20 participants, exhibited higher levels of protein carbonyls in comparison to the healthy control group, which consisted of 18 individuals (Žitňanová et al. 2006). Conversely, the study found that the antioxidant capacity was comparable between the two groups. The primary focus of this investigation was to assess the hypothesis that individuals with T21 exhibit an elevated oxidative stress status (Žitňanová et al. 2006). Likewise, Kamatsu et al. conducted an assessment of the concentration of 8-hydroxy-2'-deoxyguanosine, an oxidative stress marker, in saliva samples obtained from individuals with T21. The findings of the study confirmed the presence of oxidative stress in T21 patients based on the elevated levels of the oxidative stress marker (Komatsu et al. 2013). In their antioxidant evaluation, He et al. discovered that the total superoxide dismutase (SOD) activity was significantly higher in the T21 group compared to healthy individuals. However, the activity of extracellular glutathione peroxidase (GPx3) was found to be reduced in the T21 group. These findings highlight the altered antioxidant enzyme activities observed in individuals with T21 compared to those without the condition (He et al. 2016). It can be inferred that oxidative stress not only contributes to congenital impairments but also has detrimental effects on various biological components (Sharifi-Rad et al. 2020). Due to prolonged exposure to oxidative stress during the prenatal period, it is postulated that a decline in the compensatory antioxidant capacity of cells may exacerbate oxidative-related comorbidities, including cognitive phenotypes. Subsequent in vivo studies have been conducted to investigate and validate these hypotheses.

### In Vivo Studies and Potential Therapies

Several DS mouse models have been developed to study the neurobiological and cognitive impairment mechanisms implicated in T21 (Gupta, Dhanasekaran, and Gardiner 2016; Meng, Wang, and Ma 2018; Belichenko et al. 2015). The Ts65Dn and Ts1Cje mouse models possess an additional small region on chromosome 16 that harbors genes orthologous to those found on triplicated human chromosome 21 (D. Hamlett et al. 2015; Marechal et al. 2019; Revilla and Martínez-Cué 2020). In their study, Ishihara et al. utilized T21 mouse models (Ts1Cje and Ts2Cje) to investigate oxidative stress. The results demonstrated an elevation in ROS concentration in the brains of mice with DS compared to those without the condition. Additionally, the expression of oxidatively modified proteins and lipid peroxidation-derived products, such as 13-hydroperoxy-9Z,11E-octadecadienoic acid and 4-hydroxy-2-nonenal, was significantly increased in the brains of the T21 mouse models. These findings provide further evidence of oxidative stress in the context of DS (Ishihara et al. 2009). The study examined various in vivo protocols aimed at antioxidant therapy. The findings demonstrated that prenatal treatment had a positive impact on brain development. However, it should be noted that despite the promising results obtained, these findings have not yet been translated into clinical trials (Keck-Wherley et al. 2011; Gardiner 2014; Najas et al. 2015; Revilla and Martínez-Cué 2020). Recent data has indicated that the administration of the antidepressant fluoxetine via prenatal infusion in Ts65Dn mice resulted in improved neuronal development in the brain (Kuehn 2016; Fayçal Guedj, Bianchi, and Delabar 2014). In this case, fluoxetine supplementation in Ts65Dn mice improved the development of brain neurons, where rapamycin and α-tocopherol in preclinical studies markedly reduced lipid peroxidation and improved cognition in preclinical DS mouse models (Duval, Vacano, and Patterson 2018; Shichiri et al. 2011). Guedj et al. conducted a study demonstrating that the administration of the antioxidant apigenin offers protection against elevated oxidative stress and imbalances in total antioxidant capacity in fetal cells derived from human amniotic fluid with T21 and in the Ts1Cje mouse model (Faycal Guedj et al. 2020; Warkad et al. 2021). This study suggests that apigenin has a pleiotropic effect, leading to the activation of pro-proliferative and pro-neurogenic factors including Mki67, nestin, Sox2, and Pax6. Additionally, apigenin appears to reduce the concentrations of pro-inflammatory cytokines, such as interferon gamma, interleukin 1A, and interleukin 12P70, by inhibiting nuclear factor-κB signaling. Furthermore, it increases the concentrations of anti-inflammatory cytokines interleukin 10 and interleukin 12P40, while also enhancing the expression of angiogenic and neurogenesis factors, such as vascular endothelial growth factor A and interleukin 7 (Faycal Guedj et al. 2020).

### Strengths and Limitations of Clinical Trial

Although there is substantial evidence supporting the occurrence of oxidative stress in individuals with T21 throughout various stages of development, clinical trials conducted thus far have not yielded significant advancements in clinical management for this condition (S. E. Lee et al. 2020). A hypothesis can be put forward that prenatal antioxidant intervention may have a positive effect in mitigating cognitive impairment. This hypothesis suggests that by identifying deregulated metabolic pathways, new diagnostic targets can be identified, allowing for the implementation of optimized treatments, including during fetal life, with the aim of providing protective effects for the brain development of individuals with T21 (Fayçal Guedj, Bianchi, and Delabar 2014). Prospective clinical studies involving large cohorts of children with T21 in the future may provide valuable insights into the impact of antioxidant therapies and diet on the clinical manifestations of this syndrome. Additionally, the potential of antioxidant interventions to slow down the progression of dementia and other neurodegenerative diseases in individuals with T21 warrants further investigation. Consequently, there is a demand for the development of new antioxidants with diverse intervention strategies. Furthermore, the intricacies of oxidative remodeling in metabolic components and pathways in individuals with T21 remain incompletely understood and require further exploration (Gardiner 2014; Lott 2012; Fayçal Guedj, Bianchi, and Delabar 2014). The utilization of a redox-modulating strategy to mitigate cognitive impairment holds promise as a novel therapeutic approach. However, a comprehensive comprehension of ROS-mediated signaling in trisomy is essential to facilitate the development of innovative therapeutic interventions (Bourgonje et al. 2020).

However, oxidative stress is just one of the pathways associated with congenital disabilities in Down syndrome DS. The absence of a comprehensive DS mouse model hinders the translation of treatments to DS patients. Consequently, predicting the dose–response relationship, potential side effects, and assessing cognitive function improvement between mouse and human models remains challenging. Overcoming these challenges through rigorous experimental design and interpretation of results in both mouse and human studies could potentially minimize the risk of clinical trial failures and contribute to the effective reduction of cognitive defects in DS treatment.

## Lipid Peroxidation

Lipid peroxidation is a cascading chain reaction wherein by-products, such as radicals derived from fatty acids, interact with other free fatty acids, resulting in the formation of lipid peroxides (LPOs) (Einor et al. 2016; Nishizawa et al. 2021; Gasparovic et al. 2013; Gago-Dominguez and Castelao 2008). Moreover, the presence of LPOs enhances the reactivity of non-radical compounds by facilitating their interaction with singlet oxygen or oxyradicals. This heightened reactivity of non-radical compounds plays a significant role in modulating the redox potential (Barone et al. 2017; Ramana et al. 2017; Juan et al. 2021; Marnett 2002). The quantification of oxidized low-density lipoprotein (o-LDL), lipid peroxide (LPO), 4-hydroxynonenal (HNE), and malondialdehyde (MDA) serves as reliable indicators of lipid peroxidation, functioning as independent biomarkers (J et al. 2020; Zhihua Liu et al. 2017a, b, c; Yuan et al. 2019). Lipids play a crucial role in the structural and functional integrity of the central nervous system, accounting for approximately 60% of the constituents found in brain cells (Barón-Mendoza and González-Arenas 2022). Existing literature suggests that lipid peroxidation plays a significant role in the development of neurodegenerative diseases. It can modulate various aspects, including the physical properties of cell membranes, secondary messenger activity, and gene expression regulation. These implications are also relevant in the context of analyzing T21 (Strydom et al. 2018). Lipid peroxidation plays a crucial role in the development of cognitive impairment, and it can act as a signal integrator that translates various signals into metabolic responses. Additionally, it has the potential to impact cellular physiology and behavior on multiple levels, thereby disrupting neurogenesis processes (Müller et al. 2009).

### Prenatal Development

Perluigi et al. identified proteins that are critically relevant in T21 pathogenesis and are involved in iron homeostasis (ceruloplasmin and transferrin), lipid metabolism (zinc-a2-glycoprotein, retinol-binding protein 4, and apolipoprotein A1), and inflammation (complement C9,a-1B-glycoprotein, collagen alpha-1 V chain) (Perluigi et al. 2011). Western blotting was performed to investigate amniotic fluid samples collected from 10 individuals with T21 pregnancy and 10 individuals with a healthy fetus, serving as a control group (Perluigi et al. 2011). The comparison of maternal plasma metabolic fingerprints between euploid and T21 pregnancies revealed lower levels of five metabolites in the T21 group. Among these metabolites, linoleamide and piperine concentrations demonstrated the highest clinical significance (Parfieniuk et al. 2018). Linoleamide, also known as linoleic acid amide, participates in the lipid metabolism and specifically contributes to lipid peroxidation processes (Parfieniuk et al. 2018; Ioannou et al. 2019; Taha 2020). The reduced concentration of piperine, known for its anti-inflammatory, antioxidant, and lipid peroxidation-protective properties, has been linked to the ineffective neuroprotective characteristics observed in individuals with T21 (Parfieniuk et al. 2018; Yang et al. 2015; Azam et al. 2022; Manap et al. 2019).

### Childhood

Research conducted on T21 children has demonstrated elevated levels of oxidative stress and lipid peroxidation. In a study conducted by He et al., it was observed that T21 children exhibited higher concentrations of MDA compared to individuals without the trisomy 21 condition (He et al. 2016). A similar finding was reported in the study conducted by Manna et al. The assessment of plasma lipid peroxidation markers, including F2-isoprostanes, F2-dihomo-isoprostanes, and F4-neuroprostanes, revealed increased levels of lipid peroxidation in the T21 group (Manna et al. 2016). These disruptions are believed to be implicated in neurodegeneration and cognitive deterioration. Barone et al. provided evidence of the involvement of beta-amyloid (Aβ) and tau hyperphosphorylation in T21 neuropathy. Additionally, the accumulation of HNE was observed in the brain of individuals with T21 compared to the control tissue (Barone et al. 2017). The accumulation of HNE is thought to have a significant impact on the disruption of metabolic pathways, leading to abnormalities in glucose metabolism, neuronal trafficking, and antioxidant responses (Barone et al. 2017).

### Potential Treatment

Highlighting the significance of oxidative stress and lipid peroxidation, a study conducted by Ordonez et al. proposed that regular exercise could potentially reduce oxidative stress and subsequent lipid peroxidation in individuals with T21. In this particular study, a 12-week training program consisting of three sessions per week was administered to thirty-one male adolescents with T21. The findings demonstrated a notable reduction in the concentration of MDA, indicating a decrease in lipoperoxidation (Javier Ordonez and Rosety-Rodriguez 2007). Pharmacological investigations have been conducted utilizing the Ts65Dn mouse model of DS. One study involved administering α-tocopherol to pregnant Ts65Dn female mice starting from the day of conception throughout pregnancy, which resulted in a reduction in cognitive impairment. Furthermore, young mice were supplemented with α-tocopherol from birth until the completion of the behavioral testing period. The findings of this study indicated that α-tocopherol supplementation led to a mitigated cognitive impairment, accompanied by decreased lipid peroxidation levels when compared to the control group (Shichiri et al. 2011). In addition, supplementation with rapamycin, an inhibitor of the mammalian target of rapamycin complex 1, in Ts65Dn mice has been shown to decrease lipoxidation-mediated protein damage. This suggests that rapamycin could serve as a potential therapeutic approach to reduce the risk of developing Alzheimer's disease (AD) in individuals with DS (Curtis et al. 2020; Di Domenico et al. 2019).

### Future Perspectives

Certain lipid metabolic pathways directly influence neuronal maturation and differentiation (Knobloch and Jessberger 2017). Hence, the prevention of lipid peroxidation may potentially lead to a decrease in cognitive impairment. However, further studies are required to assess the impact of such interventions. Currently, no clinical trials have been conducted specifically targeting the reduction of lipid peroxidation through appropriate supplementation. It could be hypothesized that substances like α-tocopherol or herbal supplements such as berberine might be considered as treatment options for prenatal or adult individuals with T21, based on promising results that could be obtained from well-controlled clinical trials (Hasanein et al. 2017).

## Mitochondrial Dysfunctions

Recent research has revealed that oxidative stress-induced mitochondrial dysfunction plays a significant role in both neurodevelopment and neurodegenerative disorders (Zana et al. 2007; Emiliano Zamponi et al. 2018; Ma 2013; Kura et al. 2019).

### In Vivo Studies

Gimeno et al. conducted a study that provided evidence of accumulated damage in mitochondria of T21 fibroblasts, which was found to be associated with an elevated oxidative stress status (Antonaros et al. 2021; Liu et al. 2017a, b, c; Gimeno et al. 2014). Subsequent studies investigating mitochondrial alterations in individuals with T21 have revealed several findings. These studies have shown that T21 is associated with reduced mitochondrial structure and vascularization, as well as decreased connectivity within the mitochondrial network, when compared to normal mitochondria (Zamponi and Helguera 2019; Pallardó et al. 2010; Izzo et al. 2017; Perluigi and Butterfield 2012). Similar structural and functional mitochondrial defects have been observed in other neurodegenerative diseases (Buneeva et al. 2020; Bose and Beal 2016; Guo et al. 2013; Singh et al. 2019). The abnormal redox potential observed in individuals with T21 is not solely attributed to the overproduction of ROS, but also to the insufficient protection provided by histones. Consequently, this imbalance contributes to a higher mutation rate in mitochondrial DNA (mtDNA) (Coskun and Busciglio 2012; Hahn and Zuryn 2019). Mitochondria are recognized as essential defenders against oxidative stress, and when there is an increased damage to mtDNA, it compromises their ability to adequately counteract the detrimental effects of ROS. As a result, the reduction of negative consequences caused by ROS becomes insufficient (Ježek et al. 2018; Picca et al. 2020; Alexeyev et al. 2013). Recently, the molecular mechanisms responsible for mitochondrial damage and energy deficits were identified and characterized in several T21-derived human cells and animal models (Picca et al. 2020). Helguera et al. conducted studies on human T21 astrocytes and neuronal cultures and observed a decrease in mitochondrial redox balance, coupled with impaired membrane potential excitability. These disturbances were found to be responsible for the adaptive downregulation of mitochondrial functions. This downregulation serves as a protective mechanism, preventing excessive oxidative damage and preserving essential cellular functions (Helguera et al. 2013). Kim et al. conducted a study that demonstrated decreased protein levels of mitochondrial complexes I, III, and V in T21 brain tissue. This reduction in mitochondrial complex proteins was associated with an increased percentage of neuronal cell apoptosis (Kim et al. 2001). Accordingly, the study performed by Coskun et al. showed that mtDNA mutations were also found in T21 brain tissues (Coskun and Busciglio 2012). Neurons rely heavily on optimal mitochondrial function as mitochondria are responsible for generating cellular adenosine triphosphate (ATP) through oxidative phosphorylation. ATP production is crucial for supporting high-energy metabolism in neurons (Ullah et al. 2021). In a study conducted by Valenti et al., using the Ts65Dn mouse model, it was demonstrated that administering the polyphenol 7,8-dihydroxyflavone (7,8-DHF) to neonatal mice at a dose of 5 mg/kg/day led to a complete restoration of brain bioenergetic dysfunction and a reduction in the levels of oxygen radicals. These findings suggest that 7,8-DHF improves mitochondrial bioenergetics and mitigates mitochondria-related neurodevelopmental abnormalities in individuals with DS (Valenti et al. 2021).

### Future Perspectives

To gain a comprehensive understanding of mitochondrial dysfunction observed during the development of T21, further exploration of fundamental mitochondrial biology is necessary. This includes investigating the mechanisms responsible for maintaining mitochondrial dynamics, eliminating defective organelles, and safeguarding the integrity of mitochondrial DNA (mtDNA) during maternal inheritance and cell division. By delving into these aspects, researchers can shed light on the underlying mechanisms contributing to mitochondrial dysfunction in T21 and potentially identify targets for therapeutic interventions (Marcovecchio et al. 2021). Preserving the connectivity of the mitochondrial network is vital as it represents a significant contributing factor to proper cellular function in individuals with T21. The integrity of the mitochondrial network plays a critical role in various cellular processes, including energy production, calcium homeostasis, and apoptotic signaling. Disruptions or impairments in mitochondrial network connectivity can lead to detrimental effects on cellular physiology and contribute to the pathogenesis of T21. Therefore, ensuring the protection and maintenance of a well-connected mitochondrial network is essential for mitigating the impact of mitochondrial dysfunction in T21(Picca et al. 2020; E. Zamponi and Helguera 2019; Valenti et al. 2018; Izzo et al. 2018). Therefore, the implementation of combined therapeutic strategies that target both mitochondrial function and antioxidative processes holds significant potential for the development of new treatment approaches in the management of conditions associated with mitochondrial dysfunction and oxidative stress, such as T21. By addressing these intertwined factors simultaneously, it may be possible to synergistically enhance cellular resilience, improve mitochondrial bioenergetics, and counteract the harmful effects of oxidative stress. Such combined approaches could involve the use of mitochondrial-targeted therapies, antioxidants, and interventions aimed at promoting cellular antioxidant defense mechanisms. The integration of these strategies has the potential to provide more comprehensive and effective treatment options for individuals with T21 and related conditions (Valenti et al. 2018).

## DS Cognitive Features

Recent study evaluating the human DS brain transcriptome showed the deregulation of genes involved in neuronal differentiation (Olmos-Serrano et al. 2016). The specific gene expression alterations have been associated with axonal myelination and altered psychomotor development in DS individuals (Ponroy Bally and Murai 2021; Olmos-Serrano et al. 2016) Table 2 substantiates these findings (Table 2).

**Table 2:** Genes implicated in DS neuronal phenotypic features

Gene	Name	Protein significance	References
*APP*	Amyloid Beta Precursor Protein	Neurite outgrowth and synaptogenesis, accumulation of amyloid-β (Aβ) peptide	Lazarov and Demars (2012), Lu et al. (2011, and Nguyen (2019)
*BACE2*	Beta-Secretase 2	β-site APP-cleaving enzyme 1 (BACE1) homologue, which may influence the onset of dementia in people with DS	Barbiero et al. (2003) and Mok et al. (2014)
*DSCR1*	Down Syndrome Critical Region 1/ Regulator Of Calcineurin 1	Encodes a gene involved in calcineurin-dependent signaling pathways (Central Neural System Development) inhibition	Lee et al. (2009) and Hoeffer et al. (2007)
*DYRK1A*	Dual Specificity Tyrosine Phosphorylation Regulated Kinase 1A	A member of the Dual-specificity tyrosine phosphorylation-regulated kinase (DYRK) family involved in regulation of gene expression, cell cycle controlling processes, formation, and maturation of dendritic spines from dendrites	Duchon and Herault (2016) and Galceran et al. (2003)
*ERBB3*	Erb-B2 Receptor Tyrosine Kinase 3	A member of the epidermal growth factor receptor (EGFR) family of receptor tyrosine kinases	Olmos-Serrano et al. (2016)
*EVI2A*	Ecotropic Viral Integration Site 2A	Complex of the cellular membrane, function as part of a cell-surface receptor	Olmos-Serrano et al. (2016)
*FABP7*	Fatty Acid Binding Protein 7	Encodes a protein that binds long-chain fatty acids and other hydrophobic ligands, the establishment of the radial glial fiber in the developing brain	Sánchez-Font et al. (2003)
*GFAP*	Glial Fibrillary Acidic Protein	Glial fibrillary acidic protein synthesis, intermediate filament family of proteins	Baburamani et al. (2020)
*MYRF*	Myelin Regulatory Factor	Membrane-bound part remains attached to the endoplasmic reticulum membrane following cleavage	Olmos-Serrano et al. (2016)
*NRIP1*	Nuclear Receptor Interacting Protein 1	Interaction with the hormone-dependent activation of nuclear receptors (modulation of transcriptional activity of the estrogen receptor)	Izzo et al. (2014)
*OPALIN*	Oligodendrocytic Myelin Paranodal and Inner Loop Protein	Central nervous system-specific myelin protein that increase myelin genes expression during oligodendrocyte differentiation	Olmos-Serrano et al. (2016)
*PLD1*	Phospholipase D1	Phospholipase selective for phosphatidylcholine	Olmos-Serrano et al. (2016)
*RTKN*	Rhotekin	Mediates Rho signaling to activate NF-kappa-B and may confer increased resistance to apoptosis	Olmos-Serrano et al. (2016)
*TAGLN2*	Transgelin 2	Smooth muscle differentiation	Sobol et al. (2019)
*TMEM63A*	Transmembrane protein 63A	Osmosensitive calcium-permeable cation channel	Moldrich et al. (2008)
*VIM*	Vimentin	Filament protein intermediation	Baburamani et al. (2020)

While many cells are able to overcome increased oxidative stress, neurons in the brain are especially vulnerable to oxidative imbalance (Wang and Michaelis 2010). The postmortem study conducted by Annus et al. revealed several characteristic features of the DS brain. These features include reduced brain weight, a lower number and depth of cerebral sulci (the grooves on the surface of the brain), enlarged ventricles (cavities within the brain), and hypoplasia (underdevelopment) of the brainstem, cerebellum, frontal lobes, and temporal lobes. These structural abnormalities are commonly observed in individuals with DS and contribute to the cognitive and neurological impairments associated with the condition. Understanding the specific brain alterations in DS can provide valuable insights into the underlying pathology and guide further research and potential interventions aimed at improving the quality of life for individuals with DS (Annus et al. 2017). Psychopathology in T21 differs from the abnormalities of other intellectual disability syndromes (Onnivello et al. 2022). Among DS individuals, increased risk of AD, as well as low-intensity behavioral and emotional disorders have been observed when compared to euploid subjects (Silverman 2007).

Accordingly, Annus et al. observed that adults with DS are able to tolerate significant cortical atrophy in the presence of amyloid without experiencing detrimental effects on their cognitive function. In this study, the brains of 46 adults with DS and 30 healthy controls underwent structural and amyloid imaging. The findings suggest that the relationship between brain structure, amyloid deposition, and cognitive function may differ in individuals with DS compared to the general population (Annus et al. 2017). This study revealed a distinct neurodevelopmental phenotype in the DS brain, characterized by a thicker cortical ribbon, particularly in the frontal and occipital lobes, and a thinner motor cortex. Additionally, compared to the general population, individuals with DS exhibited disproportionately larger putamina and smaller hippocampi, which likely result from abnormal brain development and maturation. The findings underscore the complexity of cognitive impairment formation and the influence of increased oxidative stress in exacerbating these disorders. In an effort to identify T21 brain regions most susceptible to oxidative stress, a study conducted by Head et al. demonstrated that T21 newborns face higher levels of oxidative stress compared to euploid individuals. Interestingly, this process promotes the survival of cellular phenotypes that are more resistant to ROS. These results shed light on the interplay between oxidative stress and cognitive outcomes in DS and provide valuable insights into potential targets for therapeutic interventions (Head et al. 2016). The study conducted by Cenini et al. established a link between oxidative damage in the frontal cortex and the presence of Aβ plaques associated with DS. The frontal cortex was examined in 70 autopsied brains, including 29 euploid subjects and 41 DS patients. The findings demonstrated an elevated concentration of soluble and insoluble Aβ peptides, as well as an accumulation of oligomers, in the frontal cortex of DS patients. This directly contributes to the development of AD. Importantly, the study also revealed a correlation between protein carbonyls, which are formed through oxidative modification induced by ROS, and amyloid levels. These results highlight the connection between oxidative stress, amyloid pathology, and cognitive decline in individuals with DS, providing valuable insights for understanding the underlying mechanisms and potential targets for therapeutic interventions (Cenini et al. 2012).

### T21-Related Neuronal Phenotype: A Bridge Toward Alzheimer Disease

T21 is characterized by brain hypotrophy and intellectual disability starting from the early life stages (Martinez et al. 2021; Takano et al. 2017; X. Lu, Yang, and Xiang 2022). This defect is caused by reduced acquisition of a neuronal phenotype and an increase in the acquisition of an astrocytic phenotype by neural progenitor cells (J. Lu, Sheen, and Shee 2013; Busciglio and Yankner 1995)*.* In patients with T21, the development of dendrites with widespread branches was not observed, even at the time of fetal life (Becker et al. 1986; Iqbal and Eftekharpour 2017). Additionally, the upregulation of the amyloid precursor protein, located on chromosome 21, leads to the accumulation of Aβ plaques, which is associated with the development of AD and, in many instances, cerebral amyloid angiopathy (Nunomura et al. 2000). The neuropathology of DS is multifaceted and exhibits significant variability. The distinctive features of the neurodegenerative process include dysregulated free radical metabolism and impaired mitochondrial function, both of which contribute to the onset of AD in individuals with DS by middle age (Lott and Head 2001). Interestingly, elevated levels of oxidative stress could also be caused by increased release of amyloid beta-peptide (Perluigi and Butterfield 2012). Numerous studies have observed that individuals with T21 exhibit elevated concentrations of Aβ in both plasma and brain tissues, and this increase has been found to be negatively correlated with patient age (Schupf et al. 2010; “Down Syndrome and Beta-Amyloid Deposition: Current Opinion in Neurology” 2004). Furthermore, in the study conducted by Anandatheerthavarada et al., it was found that Aβ specifically targeted the mitochondria of cortical neuronal cells and certain regions of the brain, leading to neuronal changes associated with AD (Anandatheerthavarada et al. 2003; Busciglio et al. 2007). Furthermore, considering the genetic similarities between DS and AD, as the genes responsible for AD are encoded by chromosome 21, it offers an intriguing area of research for understanding many unresolved issues. Given the shared neuropathology, clinical presentation, and risk factors between DS and AD, their cognitive profiles also exhibit similarities. In a study conducted by Dick et al., it was discovered that the neuropsychological profiles of participants with DS and AD were remarkably similar (Dick et al. 2016).

## Improving T21-Related Cognitive Impairments

### In Vitro Studies

Currently, only a limited number of therapeutics and nutraceuticals have been examined using the Ts65Dn mouse model, which represents DS, with the objective of enhancing learning and memory functions (Galati et al. 2018; Choong et al. 2015; Faycal Guedj et al. 2020). Nevertheless, these studies are characterized by many limitations (Das and Reeves 2011; Rueda et al. 2012). First of all, orthologs of chromosome 21 genes map to segments of three mouse chromosomes, Mmu16, Mmu17, and Mmu10 (Gupta et al. 2016). Furthermore, it is challenging to identify dosage-sensitive genes linked to DS-related phenotypes using mouse models. As a result, DS phenotypes may be attributed to the dysregulated expression of extensive chromosomal domains across the entire genome (Antonarakis et al. 2020). Nonetheless, mouse models that encompass a broader representation of the genetic basis for DS can be valuable in enhancing our understanding of the molecular mechanisms underlying the diverse clinical characteristics of DS and ultimately leading to advancements in brain function (Das and Reeves 2011).

In a study conducted by Zhu et al., the pharmacological suppression of the integrated stress response (ISR) gene resulted in a reduction in congenital impairments. The research aimed to inhibit the ISR-inducing double-stranded RNA-activated protein kinase by enhancing the function of the eukaryotic translation initiation factor 2-eukaryotic translation initiation factor 2B complex (eIF2-eIF2B). This intervention facilitated proper translation and synaptic transmission, leading to increased synaptic activity. By silencing the ISR, significant improvements were observed in the formation of long-term memories in mice with DS (Zhu et al. 2019). Building upon the previously published studies that focused on the selective suppression of mitochondrial complex proteins and the reduction of oxidative stress through ISR silencing, additional research holds the potential to integrate multiple disrupted pathways. This would contribute to a comprehensive understanding of the biological effects of gene inhibition in DS, potentially paving the way for the development of gene therapy interventions (Zhang et al. 2021). It can be hypothesized that by comprehending the neuroanatomical features of the DS brain and implementing tailored therapeutic approaches, it may be possible to mitigate cognitive impairment and enhance cognitive functions.

Furthermore, various studies have explored strategies to enhance neurological functions in T21 mice through the promotion of neurogenesis. In a study conducted by Xu et al., the effectiveness of hyperbaric oxygen (HBO) therapy was evaluated using rat middle cerebral artery occlusion. The results demonstrated neuroprotective effects and improvements in neuronal function (Xu et al. 2016). HBO treatment was found to stimulate neurogenesis and enhance neurofunctional recovery at day 42. These beneficial effects were attributed to a reduction in ROS synthesis, and when ROS levels were decreased, the observed improvements were reversed (Ahmadi and Khalatbary 2021). Profiling additional neurogenesis factors could facilitate the advancement of medical treatments for T21. It is possible to hypothesize that inhibiting ROS in T21 pregnancies could lead to improved fetal brain development (S. E. Lee et al. 2020; Revilla and Martínez-Cué 2020). Additional research and clinical trials are needed to validate this therapeutic strategy in humans, which could initiate T21 prenatal therapy.

### In vivo studies

In vitro studies provide a basis for future clinical trials in humans. Subsequent studies on children with DS, in which α-tocopherol was supplemented at a dose of 400 IU/day, showed a significant decrease in urinary 8-hydroxy-2'-deoxyguanosine (8OHdG) concentrations. This suggests that α-tocopherol supplementation in the diets of children with DS may alleviate oxidative stress at the DNA level (Mustafa Nachvak et al. 2014). Table 3 summarizes the most crucial reports of cognitive impairment improvement and the available data from completed clinical trials (Table 3).

**Table 3:** The clinical trials among DS individuals

Study type	Participants	Type of intervention	Outcomes	References
Clinical Trials—Children	Study Group—57 Placebo Group—56	Selenium 10 μg, zinc 5 mg, vitamin A 0.9 mg, vitamin E 100 mg, and vitamin C 50 mg, folinic acid 0.1 mg	No improvement in the psychomotor and language development	Ellis (2008)
Clinical Trials—Adults	Tudy Group—26 Placebo Group—27	900 IU of α-tocopherol, 200 mg of ascorbic acid and 600 mg of alpha-lipoic acid	Improvement in cognitive functioning	Lott et al. (2011)

Future therapeutic testing should investigate whether a single-target therapy or combination therapy targeting oxidative stress is sufficient to overcome T21 cognitive impairment.

## Future Perspectives

### DYRK1A: Is the Molecular Signature of DS Phenotype Revealed?

In light of the challenges in drug development, genetic validation of drug targets has gained popularity, particularly in the management of complex trisomy disorders. Among the candidate genes associated with the characteristic phenotype of individuals with T21, DYRK1A, located on DSCR1, has emerged as the most promising target.

#### DYRK1A Inhibition—In Vitro Studies

To date, the inhibition of DYRK1A has shown the most promising results; however, several limitations still exist (Hibaoui et al. 2014). Garcia-Cero et al. demonstrated in a Ts65Dn model that increased expression of the DYRK1A gene plays a crucial role in various neurodegenerative processes observed in DS (García-Cerro et al. 2017; W et al. 2014). The overexpression of *DYRK1A* in T21 brains may contribute directly to early-onset neurofibrillary degeneration through tau hyperphosphorylation and indirectly through the phosphorylation of alternative splicing factors (Wegiel et al. 2011). Another recent study revealed the modulatory effects of natural polyphenols found in green tea on the DYRK1A kinase (Muchová, Žitňanová, and Ďuračková 2014). Consequently, DYRK1A inhibition has shown beneficial effects in mouse models of DS, including improvements in cognitive behavior. Pharmacological inhibition of DYRK1A has been achieved through treatment with epigallocatechin gallate (EGCG), yielding positive results in terms of alleviating cognitive impairment in transgenic mice overexpressing Dyrk1a and Ts65Dn mice (Hibaoui et al. 2014; J et al. 2020; Souchet et al. 2015; Stagni et al. 2016).

#### Clinical Trials of DYRK1A Inhibitor

In the first clinical study, DS patients (n = 31; aged 14–29 years) were enrolled and supplemented with EGCG (oral dose of 9 mg/kg/day) or placebo over a 3-month period. The intervention with EGCG showed improvements in visual recognition memory, working memory performance, psychomotor speed, and social functioning in individuals with T21(De la Torre et al. 2014). In the second clinical study, DS patients (n = 84; aged 16–34 years) were included, and the impact of cognitive training combined with EGCG supplementation (oral dose of 600 mg/day for participants weighing 50–75 kg or 800 mg/day for participants weighing 75–100 kg) was evaluated over a 12-month period. The combination of EGCG treatment and cognitive training resulted in improvements in visual recognition memory, inhibitory control, and adaptive behavior in the study group. Neuroimaging analysis also demonstrated enhancements in functional connectivity and cortical excitability (de la Torre et al. 2016; Feki and Hibaoui 2018). There is strong evidence supporting the involvement of DYRK1A not only in cognitive deficits associated with T21 but also in Alzheimer's disease (AD). Similar factors related to neuroinflammation and oxidative damage have been studied in both DS and AD individuals, leading to the early onset of dementia observed between the ages of 30 and 39(Becker et al. 1986; Hempel et al. 2015; Galceran et al. 2003). *DYRK1A* clearly plays a significant role as a common neurodegenerative factor, and therefore, studying the pathogenesis of DS will also contribute to the understanding of the pathogenesis of other neurodegenerative diseases.

#### Clinical Trials of DYRK1A Inhibitor Limitation

Despite the promising results obtained during in vivo studies, further DS treatment protocols aiming to inhibit the *DYRK1A* expression have been not proposed or recommended (Fayçal Guedj, Bianchi, and Delabar 2014; Faycal Guedj et al. 2020). Accordingly, due to limited data, it is still challenging to draw definitive conclusions about the effectiveness of pharmacological interventions for cognitive decline in individuals with DS. However, it is not possible to selectively inhibit *DYRK1A* expression. Therefore, EGCG acts as a mimic of multiple kinase inhibitors simultaneously (Bain et al. 2007). Moreover, the EGCG used in clinical trials is typically supplemented as green tea extracts, which contain EGCG along with various other compounds, including other catechins. Additionally, in the review conducted by Long et al., concerns regarding potential side effects and the lack of effectiveness, particularly due to flawed research planning, have been discussed (Long et al. 2019).

## Discussion

Neuropharmaceutics represents the most promising avenue for addressing cognitive impairments among DS patients, making it the largest potential growth sector in the pharmaceutical industry (Pardridge 2007; Capsoni and Cattaneo 2022). Due to the brain's effective protection by the blood–brain barrier (BBB), the administration of pharmacological agents with intracerebral biological activity poses a challenge. In this regard, intranasal delivery emerges as a viable solution in neuropharmaceutics. This approach enables the direct delivery of medications to the brain, bypassing systemic effects and potential barriers associated with the BBB (Stagni et al. 2015). Interesting findings have emerged from studies conducted by Capsoni et al. and Rosenbloom et al., highlighting the potential of intranasal delivery as a therapeutic approach for DS patients. In Capsoni et al.'s study, intranasal administration of human nerve growth factor painless (hNGFp) was performed in Ts65Dn mice at an early stage, before the accumulation of amyloid precursor protein (APP) leading to neurodegeneration. This treatment effectively reduced astrogliosis, dystrophic microglia, and promoted neurogenesis, demonstrating a potent neuroprotective effect on early DS phenotypic deficits and dementia (Capsoni and Cattaneo 2022). In the study conducted by Rosenbloom et al., the effects of intranasal insulin application were evaluated in DS patients aged 35 years and above. The findings of this study indicated that the treatment was safe and well-tolerated among DS patients, and it showed potential for cognitive improvement and memory retention. However, further research with larger study cohorts is still necessary to establish the effectiveness of this approach (Rosenbloom et al. 2020).

## Conclusions

Studies investigating the impact of oxidative stress on the occurrence of congenital fetal impairments in T21 patients have revealed a complex interplay of multiple metabolic pathways. Despite extensive research, it has not been possible to determine the potential of antioxidant supplementation in reducing cognitive impairment in T21 individuals. However, the integration of various oxidative stress-related pathways holds promise for the discovery of novel therapies in the future. In the coming decades, the challenges in the field of T21 will involve not only identifying effective therapies to mitigate cognitive defects but also understanding the molecular and cellular mechanisms underlying the characteristic phenotype resulting from genetic triplication. Exploring multitargeted antioxidant interventions in DS individuals will provide insights into the molecular and cellular mechanisms governing their nervous system function, leading to the discovery of potential therapies to overcome cognitive impairment. Among the clinical trials conducted to date, the inhibition of DYRK1A combined with cognitive training has shown significant improvements in visual recognition memory, inhibitory control, and adaptive behavior in adults with T21. Moreover, novel techniques such as intranasal delivery have been evaluated to improve cognitive impairments in DS individuals, with promising results. However, further studies in this field are still required.

## Data Availability

All data generated or analysed during this study are included in this published article.
